# Nature inspired optimization of IoT network for delay resistant and energy efficient applications

**DOI:** 10.1038/s41598-025-95138-z

**Published:** 2025-03-22

**Authors:** Gagandeep Kaur, Vipin Balyan, Sindhu Hak Gupta

**Affiliations:** 1 Department of Electronics and Communication Engineering, Amity University, Sector-125, Noida, India; 2 Department of Electrical, Electronics and Computer Engineering, Cape Peninsula University of Technology, Cape Town, South Africa

**Keywords:** Duty cycle, Golden ratio, IoT, LoRa, Optimization, PSO, Energy science and technology, Engineering

## Abstract

LoRa being an open standard has fascinated the research community due to its promising features to support IoT applications. LoRa fulfils all the requirements of low power, delay tolerance, long transmission range and scalability of the application nodes in the IoT concept. The duty cycle limitations imposed by LoRaWAN hinder the overall performance of the network. The network performance declines due to increasing in several devices communicating through the same channel, thereby degrading the network efficiency. Certain IoT deployments such as monitoring and control applications require low latency and extended network lifetime. Aspiring to attain efficient network performance, the current work proposes a nature-inspired low duty cycle MAC algorithm using the concept of the golden ratio (GR) approach to optimize the duty cycle of the LoRa network. Further, PSO algorithms have also been utilized to validate the performance of the proposed algorithm. The simulation results unveil that the proposed method outperforms the PSO algorithm by reducing the latency and power consumption by 26% and 12% respectively and extending the network lifetime by 14% as compared to the DC constraint approach.

## Introduction

The Internet of Things (IoT) is transforming our everyday experiences, offering groundbreaking technology that enhances convenience and connectivity in ways we’ve never imagined. IoT aims to seamlessly link billions of low-cost devices, enabling them to interact with one another through a unified interface, all without the need for human involvement^1^. Even in the middle of the global Covid-19 pandemic, IoT eminence in 2021 has congregated momentum from industrial IoT devices to basic healthcare safety applications and facilitating new smart city initiatives. The key requirements of IoT end devices include low power consumption and a long communication range. Certain IoT deployments such as industrial monitoring and control, health monitoring and seismic activity monitoring require low power consumption and lower latency. In such applications, immediate detection and action are required as a small delay can be disastrous. Also, in a typical IoT application, there are a massive number of connected IoT devices that are battery operated and frequent battery replacement upon depletion is challenging and can raise the operational cost of the IoT network. Such challenges can hinder the performance of the network. Low Power Wide Area Network (LPWAN) technologies are rapidly emerging as a vital solution for mission-critical IoT applications, effectively meeting demands for extensive coverage, energy efficiency, reliable connections, massive device connectivity, and consistent latency. LPWAN technologies effectively combine low power consumption with impressive long-range capabilities, perfectly bridging the divide between short-range local area networks (LAN) and extensive cellular networks^2^. LPWAN technologies embrace numerous realizations and protocols, both proprietary and open source, sharing familiar characteristics of long battery life and wide area coverage. The key contenders dominating the LPWAN market include Narrow Band IoT (NB-IoT), Long Term Evolution (LTE-M), Sigfox, Weightless, and Long-Range (LoRa) technologies^3^. Among these LoRa developed by LoRa Alliance is one of the most endorsed technologies^4^. It is explicitly targeted at battery-operated devices and offers effortless interoperability amid IoT solutions without any requirement for complex infrastructural installations^5^. A crucial aspect of the Long-Range Wide-Area Network (LoRaWAN) protocol is its implementation of duty cycles, which regulate the use of unlicensed Industrial, Scientific, and Medical (ISM) bands. This mechanism is vital as it directly influences the network’s latency limitations.

To achieve the vital balance between latency, network lifespan, and the unique requirements of IoT deployments, it is essential to develop innovative strategies that boost network performance. Optimization plays a critical role in ensuring the network operates at its highest potential. The purpose of optimization is to attain optimal performance with a set of constraints. There are numerous available tools and techniques to optimize the network. In recent decades, nature-inspired optimization algorithms have gained significant traction in addressing a variety of complex engineering challenges. These innovative algorithms draw inspiration from natural processes, mimicking how nature adapts and evolves to find effective solutions to difficult problems. The substantial success of these algorithms lies in their capabilities to attain the optimal solution within a practical period. Some of the most popular and well-adapted nature-inspired optimization algorithms are particle swarm optimization (PSO) algorithm^6^, genetic algorithm (GA)^7^, cuckoo search (CS) algorithm^8^, ant colony optimization (ACO) algorithm^9^ and many more. Out of these PSO is the simplest and most popular metaheuristic algorithm with very few customizable boundaries^10^.

Besides plants and creatures, phenomena from chemistry, physics and mathematics have also been utilized in the formulation of novel optimization approaches. There are numerous existing mathematical constants such as Archimedes’ Constant (*Pi*), Euler’s number (*e*), golden ratio ($\varphi$) and many more that have fascinated the minds of researchers due to their remarkable physical phenomena. Drawing on Archimedes’ principle from the laws of physics, Archimedes optimization algorithm (AOA) has been suggested in^11^ to resolve numerical optimization problems. Using the concept of the golden ratio, authors in^12^ have introduced the golden ratio optimization method (GROM). The Golden Ratio method is a one-dimensional search technique known for its superior convergence and stability^13^. Nematollahi et al.^14^ compared the GR method with other multi-dimensional optimization techniques such as PSO, ACO, and GA. Their study showed that the GR method, despite being based on a one-dimensional search, exhibited superior convergence rates and better stability in solving complex optimization problems. This comparison confirmed that GR can outperform or complement multi-dimensional techniques in certain contexts, especially when precise adjustments are needed for critical parameters like the duty cycle in IoT networks.

The effectiveness of the GR optimization technique has been further supported in^15^ where the GR method was used to identify influential users in social media networks. Their results demonstrated the GR method’s ability to efficiently converge, proving its capability to address real-world optimization challenges, even in high-dimensional contexts. Similarly, work in^16^ shows the application of GR in environmental trade-off optimization, further expanding its applicability beyond traditional optimization problems. These studies underscore the versatility and effectiveness of the GR method, both in one-dimensional and multi-dimensional contexts, validating its use in our work for optimizing the duty cycle in LoRaWAN networks.

Taking inspiration from this the current work considers the golden ratio constant to optimize the duty cycle of the LoRa network as it falls within the permissible limits of regulated duty cycle restriction. To enhance energy efficiency while simultaneously addressing delays caused by duty cycle limitations, a low duty cycle medium access control (MAC) algorithm utilizing the Golden Ratio (GR) approach has been proposed. Further, PSO algorithms have also been utilized to validate the performance of the proposed algorithm.

The primary impact of this work can be articulated as:

1. Mathematically modelled and evaluated the LoRa network performance in terms of latency, power consumption and lifetime
2. Proposed a nature-inspired low-duty cycle MAC algorithm based on the GR approach to optimize the duty cycle (DC).
3. Employed the PSO algorithm to validate and compute optimal duty cycle value for comparison with the proposed method.
4. Conducted an in-depth comparative analysis of optimized network performance, highlighting the advantages of the proposed approach.

The remainder of the paper is structured as follows: "Related work" section discusses related work, while "Long range (LoRa) overview" section offers an overview of LoRa technology. "System model" section frames the system model to evaluate network performance. In "Nature inspired optimization" section, the proposed low-duty cycle MAC algorithm based on the GR approach is presented. "Results" section showcases the simulation results, followed by the conclusions in "Discussion" section.

## Related work

LoRa network can be customized as per the IoT application requirements by configuring the deployed LoRa device. This allows us to use the available bandwidth wisely and optimize the network performance. A decisive investment has been made to elevate network performance, leading to impressive advancements. Duty cycle (DC) restriction impacts the performance of the network. Authors^17^ proposed an analytical model under duty cycle restrictions to evaluate LoRaWAN network performance, offering a foundation for further optimization to enhance QoS requirements. In^18^ authors have used the reinforcement learning approach to reduce the latency in AMI communication in LPWAN. Authors^19^ proposes a multi-hop LoRa network protocol designed to minimize latency effectively. To estimate the sensor node’s lifetime, an energy consumption model has been developed for approximation in^20^. Utilizing these models enables us to optimize the energy consumption of sensor nodes, thereby extending their overall lifetime.

The study presented in^21^ established an ideal transmission configuration designed to significantly enhance network performance, focusing on both throughput and energy efficiency. The authors^22^ put forward an innovative algorithm within the MAC protocol, aimed at thoroughly assessing system performance concerning throughput, latency, and energy consumption. This evaluation is essential for optimizing efficiency and enhancing overall performance.

The comparison of related work is summarized in Table 1. It reveals that several models have been proposed to assess the LoRa network performance, specifically focusing on latency, energy consumption, and the overall lifetime of sensor nodes. But these performance metrics had not been analyzed altogether. This work attempts to fill this gap. Numerous optimization techniques had been introduced in existing studies to improve the network performance. But no optimization technique has been able to resolve all the optimization problems altogether. With this motivation, a new optimization technique inspired by nature has been introduced using the golden ratio (GR) approach.

**Table 1:** Comparison of related work.

References	Approach	Performance metrics	Optimization	Trade-off/interaction
^17^	Investigated the network performance under duty cycle constraints	Latency	×	Focused only on latency, without considering how it affects power consumption or network lifetime
^18^	Employed reinforcement learning approach	Latency	✓	Optimized for latency but ignored power consumption and network lifetime trade-offs
^19^	Proposed multi-hop LoRa network protocol to minimize latency	Latency	×	Latency-focused, no consideration for power consumption or network lifetime
^20^	Developed an energy consumption model	Energy Consumption Network Lifetime	×	Modelled energy consumption and network lifetime, but latency was not considered
^21^	Derived optimal transmission configuration through bounding technique	Throughput Power Consumption Network Lifetime	✓	Optimized throughput, power consumption, and network lifetime without addressing the impact on latency
^22^	Proposed MAC algorithm	Throughput Latency Network Lifetime	✓	Focused on throughput, latency, and network lifetime without considering the power consumption trade-offs
^23^	Proposed an algorithm to determine optimized transmission parameters setting	Energy Consumption	✓	Optimized energy consumption but did not address how this affects latency or network lifetime
^24^	Optimized LoRa transmission parameters to achieve high quality of service	Latency Energy Consumption Throughput	✓	Focused on latency, energy consumption, and throughput, ignoring their interactions with network lifetime
^25^	proposed a scheme for combined distribution of spreading factors and transmission power with the Gurobi optimization solver	Network Energy consumption Throughput Data Extraction Rate	✓	Optimized energy consumption and throughput, but does not address latency or network lifetime trade-offs
^26^	Proposed MAC layer that leverages local packet damage and changing channel conditions	Latency	×	Latency-focused without considering power consumption or network lifetime
^27^	Formulated the problem as minimizing the transmit power of EDs while taking into account per-ED and sum-ED data rate constraints	Power Consumption	✓	Optimized power consumption but ignored the impact on latency or network lifetime
Current work	Proposed an algorithm based on golden ratio approach	Latency Power Consumption Network Lifetime	✓	Holistically optimizes latency, power consumption, and network lifetime by balancing all three metrics through duty cycle optimization. Interactions between these metrics are considered and optimized simultaneously

## Long range (LoRa) overview

LoRa is the leading LPWAN technology developed by Semtech Corporation, gaining traction among scientific and industrial communities due to its potential to enhance global IoT deployment coverage^4^. As a physical layer technology, it uses chirp spread spectrum (CSS) modulation scheme, that facilitates reliable communication over long distances within the sub-GHz ISM bands of 433, 867, and 915 MHz, depending on the operating region. LoRa leverages this modulation technique through five adjustable parameters: spreading factor (SF), bandwidth (BW), coding rate (CR), transmission power, and carrier frequency^23^. LoRa provisions seven spreading factors from 7 to 12, with SF7 providing the highest data rate and SF12 the lowest. The LoRa network operates at bandwidths of 125 kHz, 250 kHz, or 500 kHz. While a higher bandwidth results in a higher transmission rate, it also introduces additional noise, which can decrease sensitivity^28^. It employs forward error correction (FEC) codes, defined by the coding rate (CR), to enhance receiver sensitivity. Each increment in the spreading factor (SF) doubles the packet transmission time while approximately reducing receiver sensitivity by 3 dB^29^. The packet transmission time, ${T}_{a}$, of the packet as presented in^28^ is denoted as

1 $${T}_{a}=\left({N}_{payload}+{n}_{preamble}+4.25\right)\frac{{2}^{SF}}{BW}.$$

where ${N}_{payload}$ is the number of symbols used to transmit a specific payload, *PL* (in bytes), as outlined in^30^ is defined as

2 $${N}_{Payload}=8+\left(ceil\left(\frac{8PL-4SF+28+16CRC-20H}{4\left(SF-2DE\right)}\right)\left(\frac{4}{4+m}+4\right),0\right).$$

Above the physical layer is the MAC layer standardized by the LoRa Alliance, known as LoRaWAN. Because it operates within the ISM band, LoRaWAN imposes duty cycle restrictions in many countries on how devices access the shared channel, which is quantified as a percentage of the time allowed for channel access^31^. Communication in LoRaWAN takes place between three components: end devices, gateways, and network servers, all connected in a star topology. LoRaWAN end devices are classified into three categories: Class A, Class B, and Class C. Class A end devices transmit messages to the gateway and, after sending an uplink, open two receive windows at delays of 1 s and 2 s to receive downlinks from the gateway. The network server plays a vital role in managing timing and scheduling downlinks with precision, ensuring optimal power consumption and enhancing overall system performance. Class B devices offer additional receive windows compared to Class A, which are synchronized through beacons sent periodically by the gateway. In contrast, Class C end devices remain in reception mode, enabling them to receive messages continuously, except during transmission, which results in higher energy consumption. While Class A and Class B devices can experience increased channel latency due to their scheduled receive windows, Class C devices enjoy lower channel latency since there are no constraints on reception.

The next section formulates a system model for a LoRa network operating in the IN 865–867 MHz band and evaluates its performance.

## System model

In this work, we consider *n* number of end devices connected to the gateway. The available bandwidth is segmented into *N* sub-channels with each sub-channel accommodating multiple devices that access the channel through the utilization of distinct orthogonal spreading factors. Since only the LoRa network is considered so no collision due to another network transmission could take place. Further to this class, A devices are assumed which transmit the message without acknowledgement and thus no retransmissions are reflected. Class A devices remain in sleep mode for the majority of the time, resulting in lower power consumption. The transmission of packets into the channel by the end device follows the Poisson distribution. The packet generation rate by a device, $\lambda$, is defined as $\lambda =\frac{{10}^{-4}}{{T}_{a}} {s}^{-1}$^18^.

Due to the usage of the ISM band, LoRaWAN imposes the duty cycle (DC) restriction in many countries. These duty cycle restrictions impact the network performance as they influence the latency and lifetime of the LoRa network. The extent of time the LoRa devices can use the shared channel is termed as duty cycle and as mentioned in [110] represented by (3.1)

3 $$DC=\frac{{T}_{a}}{{T}_{tx}}=\frac{{T}_{a}}{{T}_{s}+{T}_{a}}.$$

where ${T}_{tx}$ is the transmission period and ${T}_{s}$ is the sleep period.

The transmission of the packets in the LoRa network are scheduled such that the end-device waits for the transmission to occur till the end of the receive window. LoRa sensor nodes are assumed to follow a fixed transmission period (*T*_*tx*_) to increase their operation which is constitutes of an active period and sleep period. Due to duty cycle (DC) restriction, the device goes into sleep period, ${T}_{s}$, after transmitting the packet and as indicated in^32^ it can be represented as

4 $${T}_{s}={T}_{a}\left(\frac{1}{DC}-1\right).$$

### Latency

The device efficiently chooses a channel at random from the complete range of available channels across all sub-bands. If none of the sub-band is available it waits in the queue for the next available sub-band. Due to duty cycle restriction, the transmission waiting time ${T}_{w}$ in each sub-band can be calculated using the queue theory. The waiting time in sub-band *i*, as outlined in^17^, can be articulated as

5 $${T}_{wi}=\frac{{P}_{busy}}{\left(\sum_{i=1}^{N}{\mu }_{i}+\lambda \right)\times 2}.$$

where ${P}_{busy}$ is the Erlang-C probability of all servers being occupied and μ denotes the service rate which is reciprocal to transmission period, ${T}_{tx}$, and as stated in^17^ is expressed as

6 $$\upmu =\frac{1}{{T}_{tx}}=\frac{1}{{T}_{s}+{T}_{a}}.$$

Transmission latency represents the total time taken to send a packet from the source to the destination. This latency encompasses processing time, packet transmission time, and propagation time. If we consider the propagation and processing times to be negligible, latency, as stated in^17^ can be expressed as

7 $${T}_{l}={T}_{a}+{T}_{w}.$$

Using the value of packet transmission time from Eq. (4), latency can be represented as

8 $${T}_{l}=\left(\frac{{T}_{s}\times DC}{1-DC}\right)+{T}_{w}.$$

There is an acute relationship between latency, battery lifetime and deployment of smart IoT applications. Frequent battery replacements can be required to uphold the continuing tasks which may be cost-effective. Thus, a prolonged battery lifetime becomes a major requirement. A precise energy model of the sensor node of the LoRa network is vital to approximate the node’s lifetime.

### Energy consumption and network lifetime

The two primary components of the sensor that consume energy are the processing unit and the transceiver unit. Energy consumption, ${E}_{c}$, during the transmission period includes energy consumed during both the sleep period and the active period by the microcontroller unit and the LoRa transceiver. As indicated in^33^, this energy consumption can be represented as

9 $${E}_{c}=\left[{T}_{a}\left({P}_{MCU\_on}+{P}_{Tx\_on}\right)+{T}_{s}\left({P}_{MCU\_off}+{P}_{Tx\_off}\right)\right].$$

Using the value of packet transmission time from Eq. (4), enengy consumption can be represented as

10 $${E}_{c}={T}_{s}\left[\left(\frac{DC}{1-DC}\right)\left({P}_{MCU\_on}+{P}_{Tx\_on}\right)+\left({P}_{MCU\_off}+{P}_{Tx\_off}\right)\right].$$

By using (10) power consumption by the sensor node can be evaluated. The study in^34^ offers us (11) to compute the lifetime of the LoRa sensor node.

11 $$Lifetime=\frac{{C}_{batt}\times {V}_{batt}}{{T}_{s}\left[\left(\frac{DC}{1-DC}\right)\left({P}_{MCU\_on}+{P}_{Tx\_on}\right)+\left({P}_{MCU\_off}+{P}_{Tx\_off}\right)\right]}.$$

Table 2 summarizes the notation used in this section.

**Table 2:** Summary of notations used.

Symbol	Definition
${n}_{preamble}$	preamble symbols (default size of 8 bytes)
*CRC*	*CRC* = *1* when enabled otherwise 0
*H*	*H* = *0* when header enabled otherwise 1
*DE*	*DE* = *1* enabling low data rate optimization otherwise 0
*m*	code rate with values (1, 2, 3, 4}
${P}_{MC{U}_{\_off}}$	power consumption of microcontroller in off state
${P}_{MCU\_on}$	power consumption of microcontroller in on state
${P}_{Tx\_off}$	power consumption of the LoRa radio unit in off state
${P}_{Tx\_on}$	power consumption of the LoRa radio unit in on state
${C}_{Batt}$	capacity of the battery
${V}_{batt}$	maximum voltage applied to the battery

The sleep period, ${T}_{s}$, plays a pivotal role in determining both latency and power consumption in LoRaWAN networks. A longer sleep period allows devices to conserve energy by remaining inactive, thereby extending battery life. However, this extended inactivity can lead to increased latency, as devices may need to wait longer to transmit data. Conversely, a shorter sleep period reduces latency but increases power consumption due to more frequent activity. To achieve an optimal balance between latency and power consumption, it is essential to determine the ideal sleep period. This involves analyzing the trade-offs between energy savings and the responsiveness of the network. By optimizing the sleep period, network performance can be enhanced, leading to more efficient and reliable communication. Therefore, in the next section, we will define an optimization problem aimed at calculating the optimal sleep time for end devices to enhance network performance efficiently.

## Nature inspired optimization

### Problem formulation

The primary goal is to determine the optimal duty cycle that minimizes the sleep period, utilizing the objective function outlined in Eq. (4), while confining ${T}_{a}$ to the maximum allowable value specific to SF. LoRa specification of IN865-867 MHz band with the BW = 125 kHz and CR = 4/5 is considered. The duty cycle for LoRaWAN ranges from 0.1 to 1%. LoRa devices operating under EU 863–870 MHz ISM band are limited to a maximum transmit duty cycle of 1%. Duty cycle restrictions as implemented on other regional bands are not instigated on IN 865–867 MHz band. Thus, the goal is to find the optimal value of duty cycle while keeping ${T}_{a}$ under ${T}_{a\_max}$ calculated using Eq. (1) for each SF. To attain this, a low-duty cycle MAC algorithm based on the GR approach has been proposed. Furthermore, the PSO algorithm is utilized to find the optimal duty cycle value. This method was selected primarily for its ease of implementation and minimal computational resource requirements.

### Low duty cycle MAC algorithm based on golden ratio

**Fig. 1:**
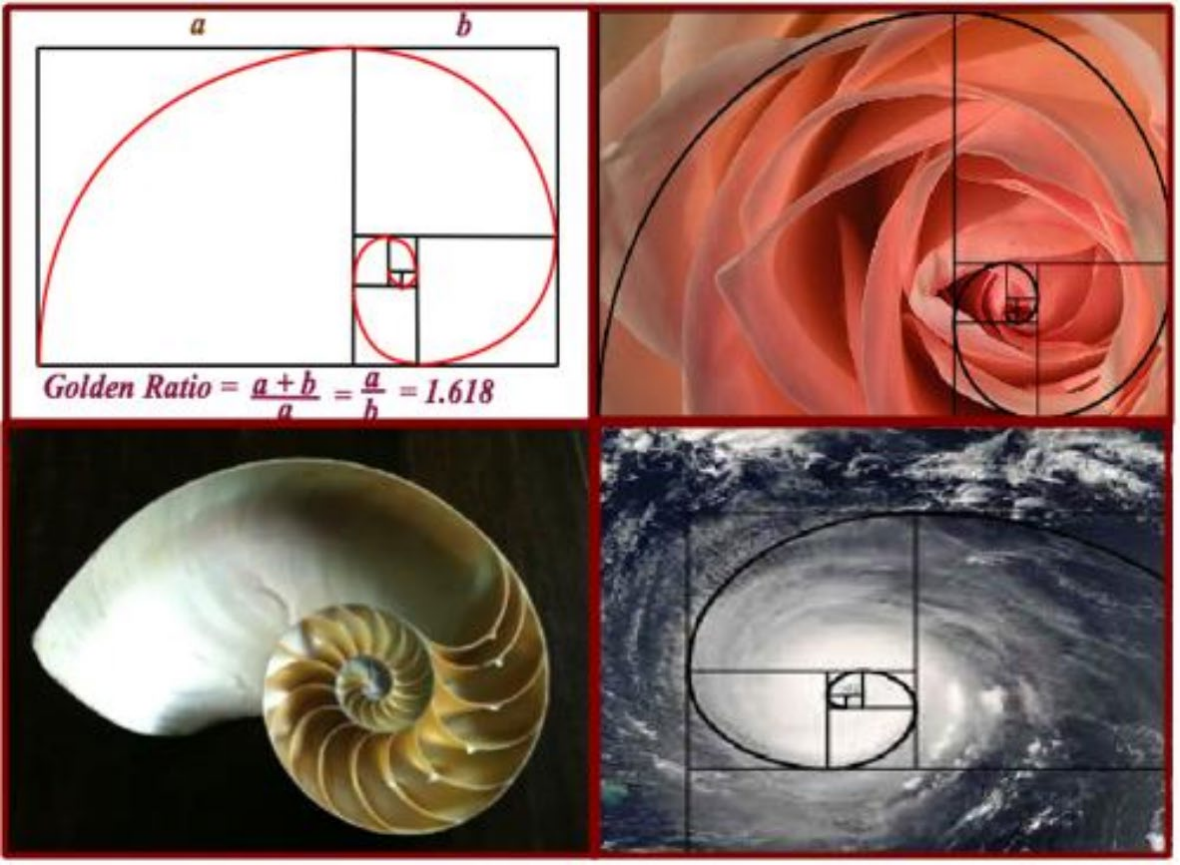
Existence of golden ratio in nature^35^.

With the advancement in human sciences, it has been recognized that everything in nature is in special order from diverse creatures to physical phenomena. The growth pattern of plants, insects, animals, and the physical phenomenon like hurricanes in this universe is found to be based on the golden ratio. Figure 1 depicts the existence of the golden ratio in nature. First introduced by the mathematician Euclid to address a mathematical dilemma, is an irrational number defined by the relationship of a line segment divided into two unequal parts. In this ratio, the sum of the longer part (a) and the shorter part (b) to the longer part is equal to the ratio of the longer part to the shorter part. It is designated by a Greek letter φ (Phi), named after Phidias, whose value is equal to the $\left(1+\sqrt{5}\right)/2=1.618033987$ (approx.)^36^. In the thirteen century the mathematician Leonardo Pisano, nicknamed Fibonacci, described a series of numbers which was later named after him^37^. As the Fibonacci series unfolds, the ratio of two consecutive integers increasingly approximates the golden ratio, highlighting a profound connection between mathematics and nature that becomes clearer with each step of the sequence. In the human body, the golden ratio is found within the extents of teeth, limbs, hands, cardiovascular system, facial features and the DNA molecule^38^. Nowadays golden ratio is used in design of industrial products which can contribute to the improvement of the visual imprint of the product on the consumer^39^. The golden ratio approach offers high accuracy while requiring low levels of differentiability and convergence for the function^13^. With its prominent features, it opens up a new and efficient way of solving various optimization problems in computational science^40^.

For efficient network performance, the LoRa network should possess reduced latency and an improved lifetime of the sensor node. Thus, to optimize these parameters concept of the golden ratio is a well-thought-out approach inspired by nature.

In this work, we propose the duty cycle variables to be in the golden ratio. The approach is to bring the duty cycle variables to be in extreme ratio as represented by (12)

12 $$\frac{{T}_{a}}{{T}_{tx}}=\frac{{T}_{a}}{{T}_{s}+{T}_{a}}=\varphi \%=1.6180 \%,$$

Solving (12) we get

13 $${T}_{s}=60.80\times {T}_{a}.$$

where φ is the golden ratio.

With this, the ratio of packet transmission period and total transmission time can be discovered to be in golden ratio satisfying (12). Algorithm 1 presents the details of presenting the duty cycle variables in the golden ratio. The algorithm works as follows. At the fixed value of payload size, SF, CR and BW the packet transmission time is generated using (1). With this calculated value of packet transmission time and the approach to take the ratio of packet transmission period and total transmission time to be in golden ratio, the value of sleep time is computed as given by Eq. (12). Each iteration generates a new total transmission time in a sequence of Fibonacci series as the sum of the previous two. By utilizing the computed sleep period, we can calculate the latency, power consumption, and sensor node lifetime. This approach enhances latency, power consumption, and the overall lifetime of the LoRa network, resulting in improved performance and efficiency.
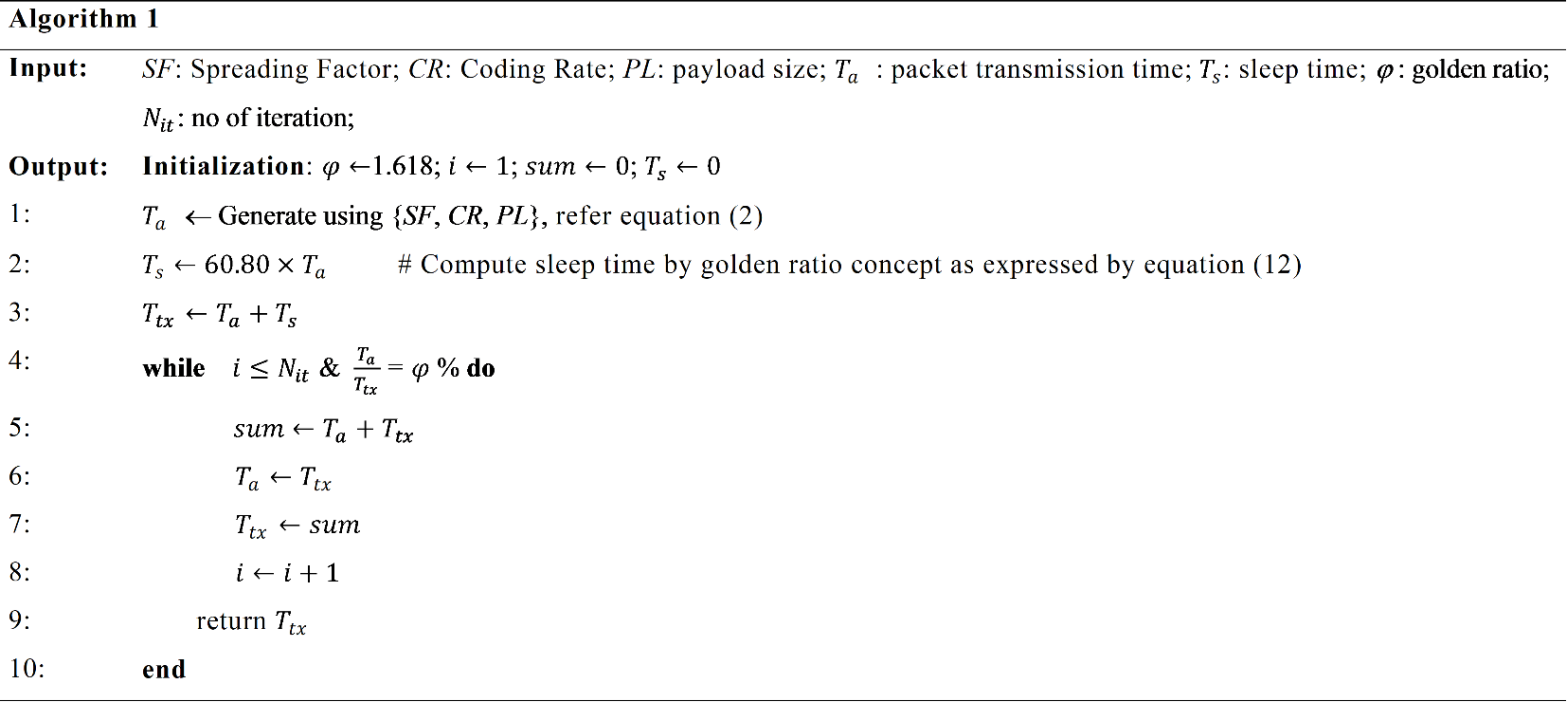


### Particle swarm optimization (PSO) algorithm

PSO is nature inspired metaheuristic optimization algorithm drawing inspiration from swarm intelligence, the algorithm seeks to identify the optimal solution to the problem by leveraging the collective experience of the entire population of particles (swarm) within the multi-dimensional search space^41^. To build a diverse and dynamic population, we begin by randomly generating the initial positions and velocities of the particles throughout the search space. This approach ensures a robust exploration of possibilities. Each particle moves towards the best fitness value by adjusting its position and velocity according to its own experience and neighborhood particles. The best fitness value corresponding to an individual particle’s position is referred to as the pbest value, while the overall best fitness value among all particles is known as the global best (gbest) value. The fitness value is achieved through the objective function that is optimized by updating each particle in the population. During each iteration, the particles accelerate toward their pbest and gbest values, with their velocity determined by assessing the distance from the target value. Algorithm 2 outlines the operational specifics and the parameters employed to optimize sleep time. Utilizing PSO algorithm, the duty cycle is optimized by the objective function described by Eq. (3). For each SF, the threshold value of packet transmission time (*T*_*a*_), is considered to be less than the calculated value obtained from Eq. (1). With the help of PSO, sleep time is minimized thereby optimizing the duty cycle up to 1.29%.
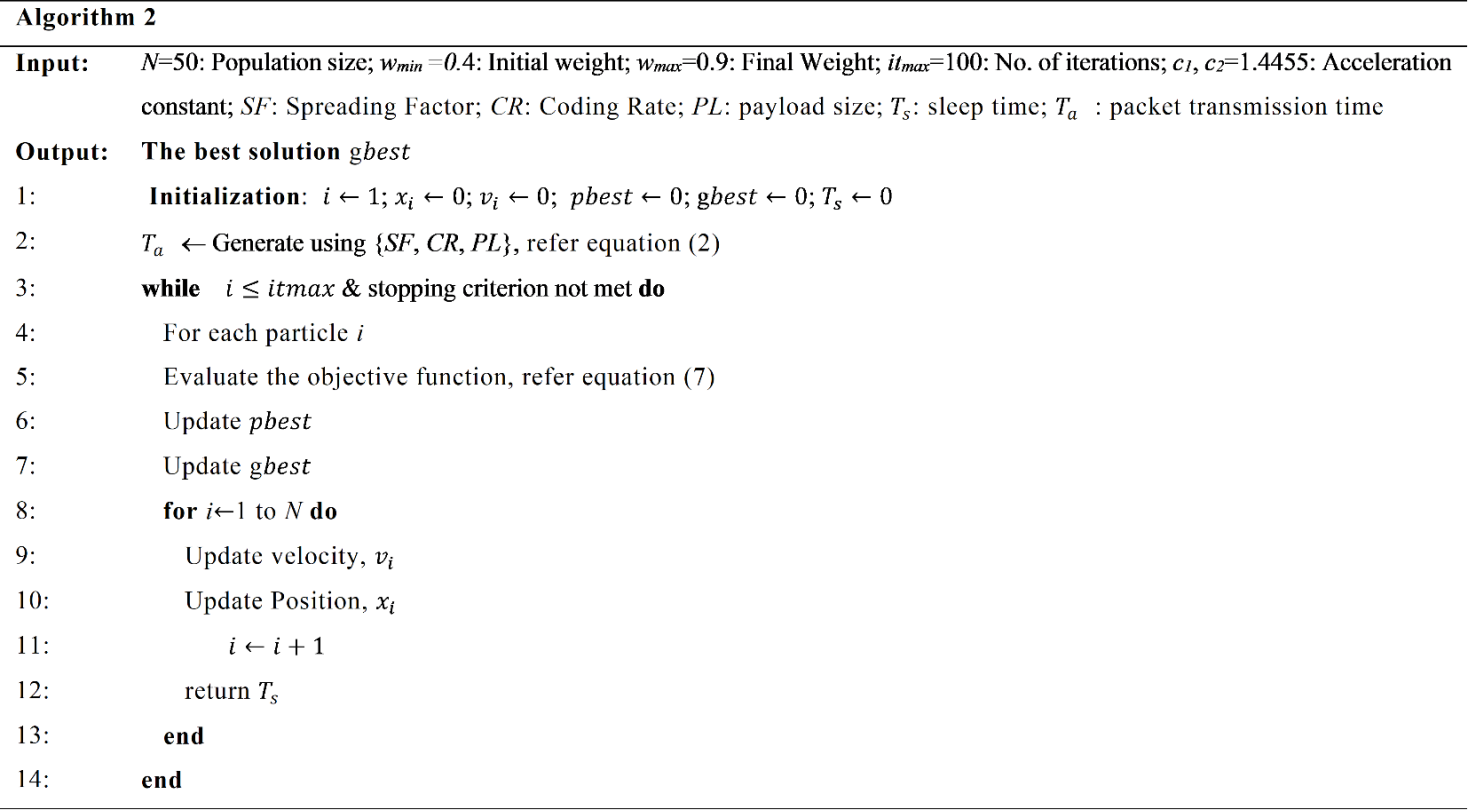


## Results

After determining the optimal duty cycle value, the network’s performance is examined focusing on latency, energy consumption, and network lifetime. The simulations were conducted using MATLAB R2019b for a LoRa network operating in the IN 865–867 MHz ISM band, focusing on latency, energy consumption, and network lifetime. The key parameters included a bandwidth of 125 kHz, as it provides a good balance between range and data rate, and a coding rate of 4/5, providing a good trade-off between reliability and overhead. The spreading factor (SF) was varied from 7 to 12 to assess the impact on range and data rate, while a default payload size of 20 bytes was selected to represent typical application data, with variations in payload size explored in some runs. The preamble symbol length was set to 8 bytes, offering sufficient synchronization space without excessive overhead. After determining the optimal duty cycle through the proposed low duty cycle MAC algorithm, the results were compared with those achieved under a default 1% duty cycle-constrained approach and PSO-based optimization. This setup enabled a comprehensive analysis of how varying these parameters influenced the network’s performance.

**Fig. 2:**
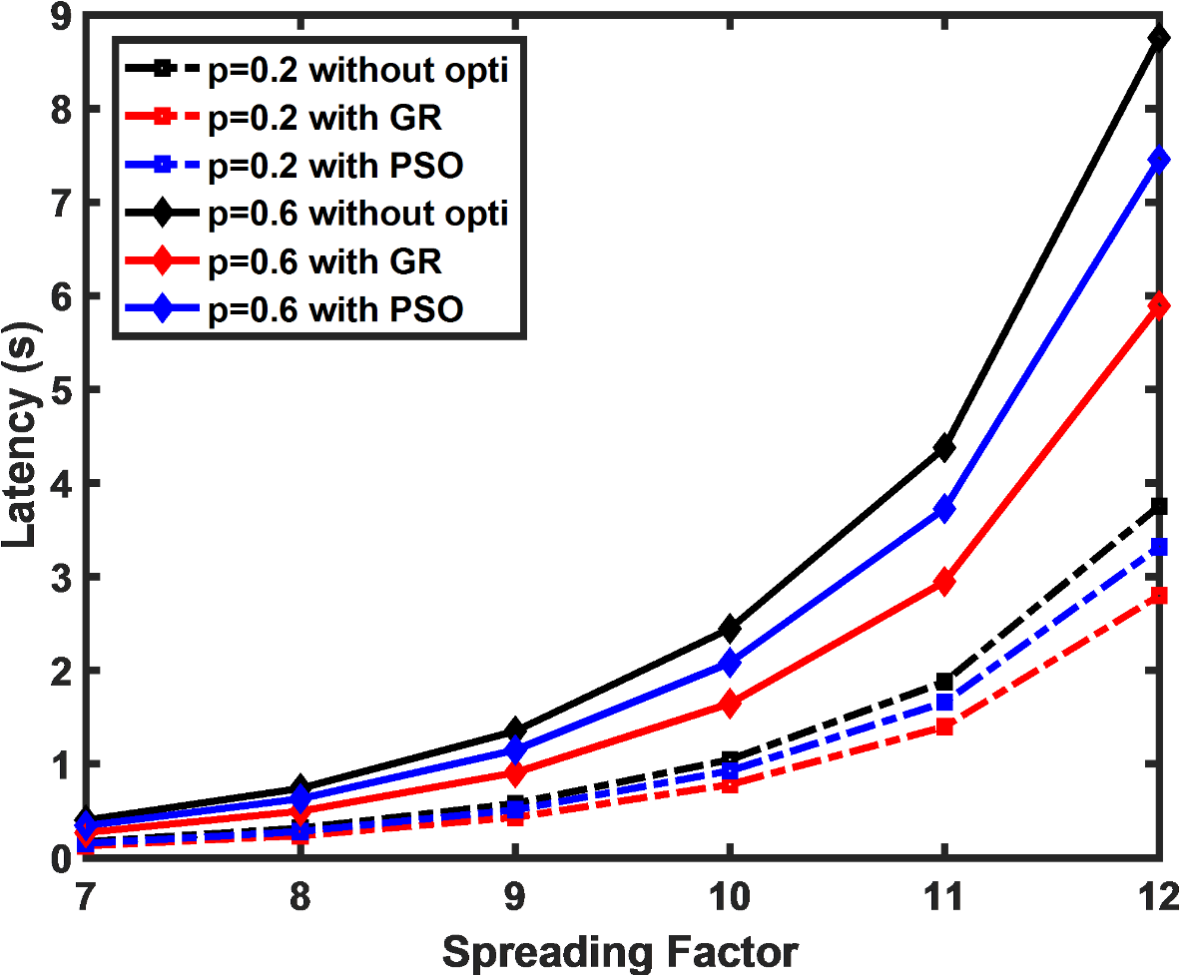
Latency decrease achieved by applying the proposed algorithm in comparison to the DC constraint approach.

Latency, as given by (6), is evaluated numerically including waiting for time restricted by duty cycle and packet transmission time with the function of SF as depicted in Fig. 2. The waiting time described in (4) directly correlates with the Erlang C probability of all servers being occupied. When examining latency across varying probabilities of server utilization, the findings reveal that reducing latency is achievable by minimizing the chance of a server being busy. This highlights the importance of managing server load to enhance overall efficiency. In the considered scenario, both the approaches, the PSO algorithm and the proposed low duty cycle MAC algorithm using the GR approach contributes to reducing the latency as depicted in the figure. The result shows that there is a decrease in average latency by 25% by applying the proposed algorithm and by 12% using PSO as compared to default DC constrained approach when the probability of the server being busy is 0.6. The figure highlights a significant decrease in latency when employing the GR approach and PSO algorithm, with the GR approach achieving a 32% reduction in average latency when the probability of server utilization is 0.2. This reduction indicates the effectiveness of the proposed algorithm in managing waiting time, which is calculated based on Erlang C probability, by reducing the likelihood of server congestion. In contrast, the PSO algorithm delivers a more modest reduction of 15% under the same conditions. This demonstrates that minimizing server load through an optimized duty cycle can substantially improve latency, underscoring the importance of dynamic resource management. The figure also illustrates that as the spreading factor increases, latency correspondingly rises.

**Fig. 3:**
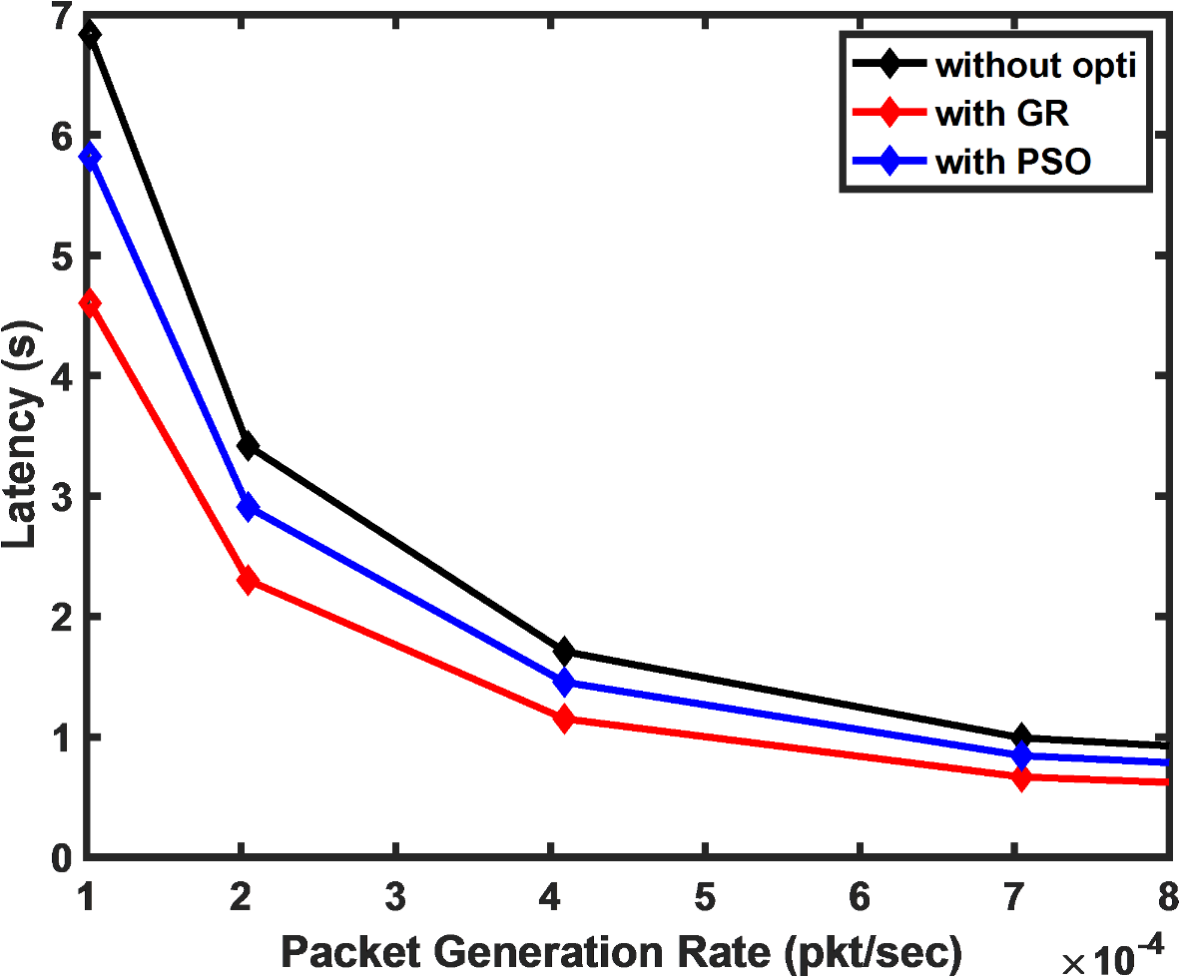
Latency versus packet generation rate at different values of the server being busy.

Figure 3 illustrates the impact of packet generation rate on latency. It is evident that as packet generation rates increase, the latency reduces exponentially. This exponential decay indicates the system’s responsiveness to packet generation, and the findings emphasize that the GR approach delivers a more significant improvement in latency reduction, especially under lower server utilization probabilities.

To compute the average consumed power by the end device, a sensor node equipped with the Arduino Pro Mini microcontroller unit with a power consumption of 12.49 mW and 81.08 µW at active state and sleep state respectively along with SX1272 LoRa transceiver unit is considered. By using (7) energy consumption by the sensor node during a single transmission cycle is numerically computed. For the simulation work, the sensor node powered by a battery of capacity, ${C}_{Batt}$, 2 mAh operating at the maximum voltage, ${V}_{batt}$, 3.3 V is assumed.

**Fig. 4:**
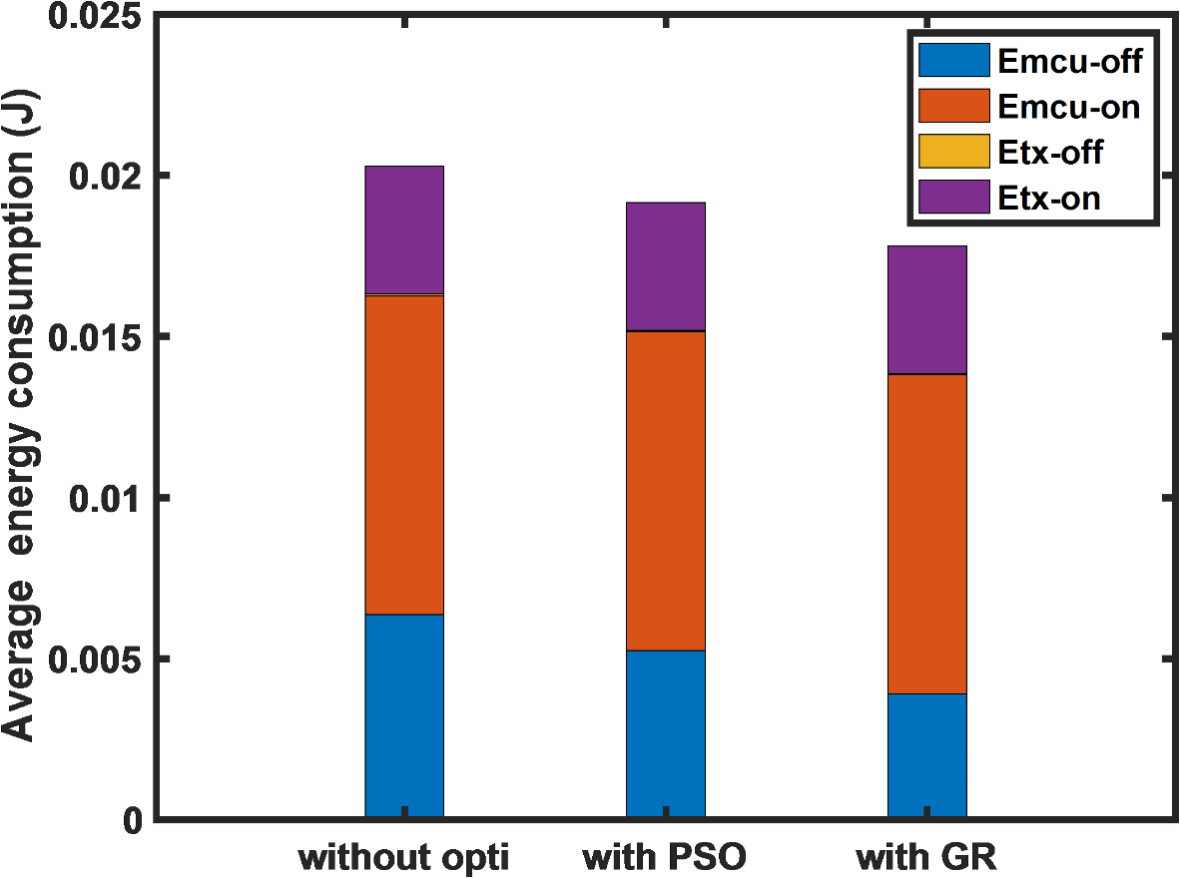
Comparative analysis of total energy consumption during different states of the sensor node.

Figure 4 offers a comparative investigation of the proposed algorithm against the PSO algorithm and the default DC constraint approach regarding the node’s average energy consumption during a single transmission cycle. The results reveal that employing both optimization strategies results in minimizing average energy consumption, as evidenced by the decline in energy usage by both the microcontroller unit and the LoRa transceiver unit while in sleep mode. The GR-based algorithm leads to a more pronounced reduction in energy usage, highlighting its efficacy in optimizing both the microcontroller unit and the LoRa transceiver’s power consumption.

**Fig. 5:**
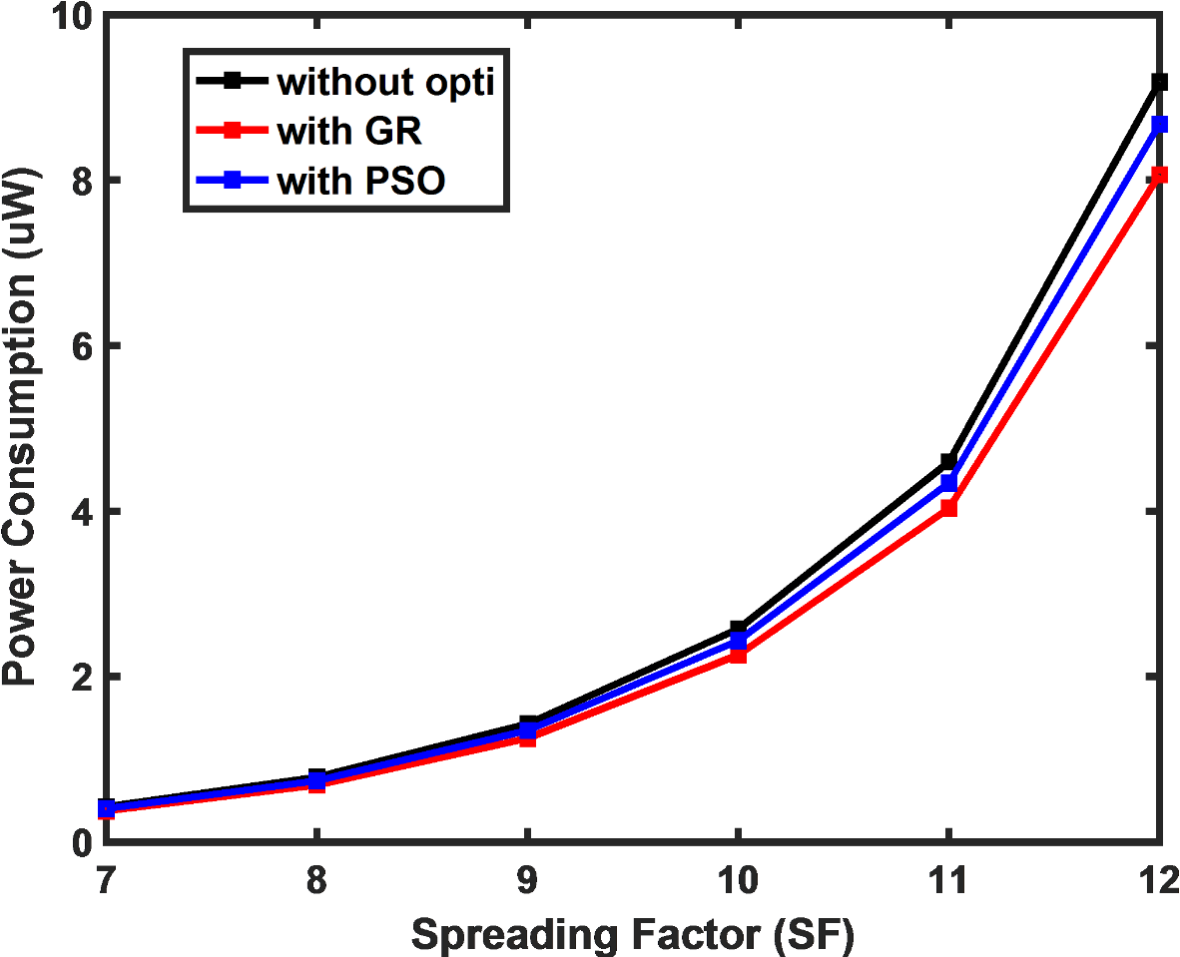
Drop in power consumption achieved using proposed algorithm in comparison to DC constraint approach.

**Fig. 6:**
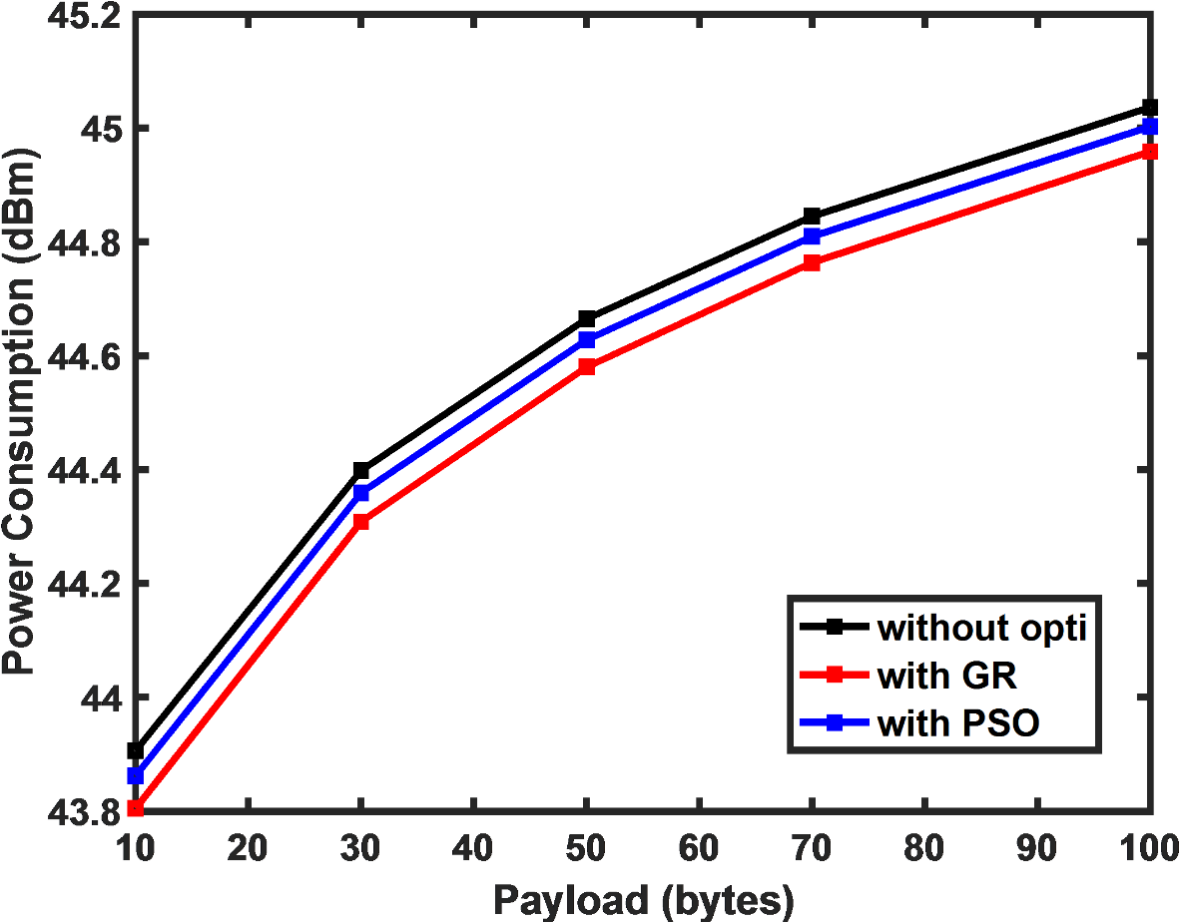
Power consumption versus payload.

Figure 5 illustrates the influence of the SF on power consumption by the LoRa end device. The results demonstrate that as the spreading factor increases, power consumption by the end device also rises, leading to a diminished lifetime for the sensor node. The figure also reveals that by applying the low duty cycle algorithm the average power consumption by the device reduces by 12% and by 6% using the PSO algorithm as compared to the default DC constraint approach. Similarly, Fig. 6 demonstrates how the payload size influences power consumption, with a clear exponential rise in consumption as the payload increases. This highlights the need for optimization techniques that balance energy usage and payload requirements, crucial for maintaining network efficiency and extending the sensor node’s operational lifespan.

**Fig. 7:**
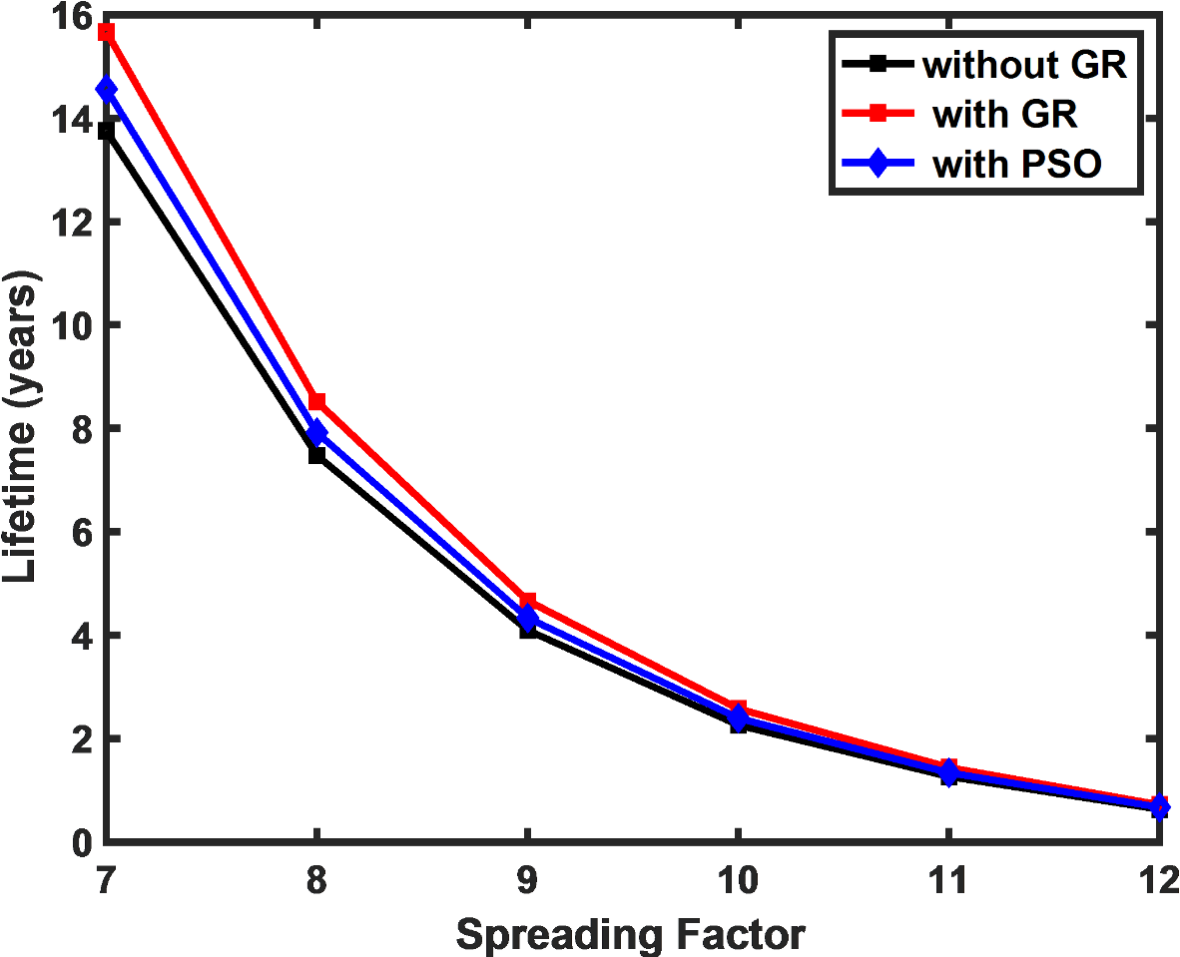
Extended lifetime achieved using proposed algorithm in comparison to the DC constraint approach.

The theoretical evaluation of the node lifetime, as given by Eq. (8), is illustrated in Fig. 7. The figure illustrates the extended lifetime of the sensor node achieved by applying the GR-based low-duty cycle algorithm compared to the default DC constraint approach. The results demonstrate a 14% increase in network lifetime with the GR approach, emphasizing the impact of duty cycle optimization on long-term performance. This figure is a key indicator of the algorithm’s effectiveness in enhancing the sustainability of LoRa networks, as it translates into reduced energy consumption and prolonged operational periods for the sensor nodes.

Furthermore, Table 3 provides a comprehensive comparison of the performance metrics of the LoRa network, both with and without optimization. This table allows for a detailed evaluation of how the proposed low duty cycle MAC algorithm impacts various network parameters, particularly latency, power consumption, and network lifetime. The table clearly highlights the improvements achieved by the optimization, giving us a direct comparison of the performance before and after the implementation of the proposed algorithm.

**Table 3:** LoRa network performance with and without optimization.

SF	Latency (s)			Power consumption (µW)			Network lifetime (yrs.)		
	WO	PSO	GR	WO	PSO	GR	WO	PSO	GR
7	0.17	0.15	0.13	0.43	0.40	0.37	13.8	14.6	15.7
8	0.32	0.28	0.24	0.78	0.74	0.69	7.5	7.9	8.5
9	0.58	0.51	0.43	1.43	1.35	1.25	4.1	4.3	4.7
10	1.05	0.93	0.78	2.58	2.43	2.26	2.3	2.4	2.6
11	1.88	1.66	1.40	4.59	4.34	4.03	1.3	1.3	1.4
12	3.76	3.32	2.80	9.19	8.68	8.07	0.6	0.7	0.7

**Fig. 8:**
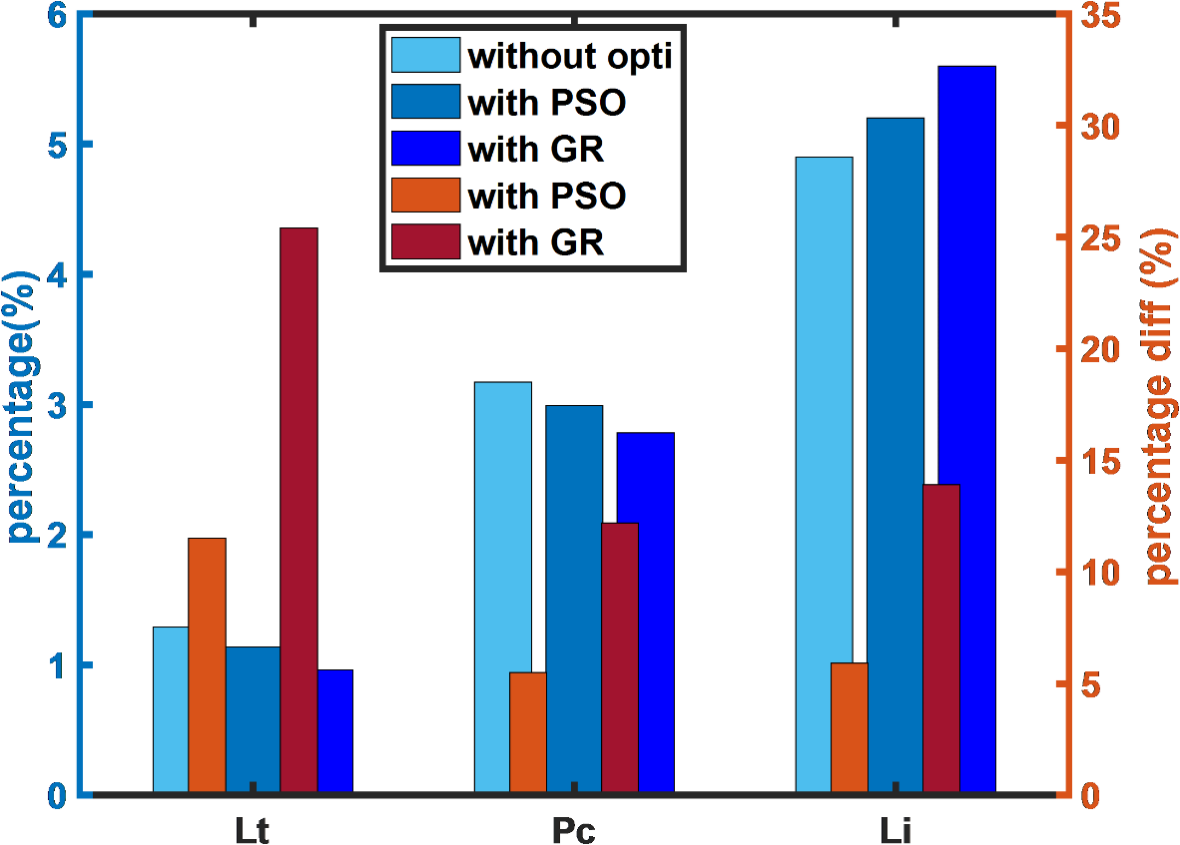
LoRa network performance: unoptimized versus optimized.

Figure 8 further supports the analysis by visually illustrating the percentage improvements across the key performance metrics. The graph emphasizes that, when the PSO algorithm is employed, latency (Lt) decreases by 12%, power consumption (Pc) is reduced by 6%, and the network lifetime (Li) is extended by 6%, compared to the default DC constraint approach. This comparison underlines the effectiveness of PSO in improving network performance, albeit with more modest improvements.

The improvement is even more pronounced when the proposed low duty cycle MAC algorithm based on the golden ratio approach is implemented. As seen in Fig. 8, the GR approach achieves a 26% reduction in latency, a 12% reduction in power consumption, and a 14% increase in network lifetime compared to the default DC constraint approach. These results clearly demonstrate the superior performance of the proposed algorithm over the PSO algorithm, making a compelling case for its application in LoRa network optimization.

This comparative analysis highlights the significance of using the low duty cycle MAC algorithm based on the golden ratio approach as an optimal solution for improving the performance of LoRa networks in terms of latency, energy efficiency, and network longevity. The clear improvements in these areas emphasize the value of the proposed algorithm in real-world network deployment scenarios.

To further solidify the results presented in Table 3, the average and standard deviation (SD) for the key performance metrics- latency (Lt), power consumption (Pc), and network lifetime (Li) for each of the optimization algorithms (PSO, and GR) has been calculated and presented in Table 4. This detailed analysis will help quantify the improvements achieved by each algorithm and will serve as a more rigorous foundation for the observations made in the comparative analysis.

- Average Calculation

**Table 4:** Average and standard deviation of performance metrics.

Metric	WO (Avg ± SD)	PSO (Avg ± SD)	GR (Avg ± SD)
Latency(s)	1.29 ± 1.24	1.14 ± 1.24	0.96 ± 0.92
Power Consumption (μW)	3.17 ± 3.02	2.99 ± 2.86	2.78 ± 2.66
Network Lifetime (yrs.)	4.93 ± 4.56	5.20 ± 4.83	5.60 ± 5.19

The average for each performance metric is computed by summing all the values for a given optimization method (WO, PSO, or GR) and dividing the total by the number of data points (SF values).

- Standard Deviation (SD) Calculation

The standard deviation quantifies the spread of the data values around the average. It will be computed using the following formula:

13 $$SD=\sqrt{\frac{\sum {\left(x-mean\right)}^{2}}{n-1}.}$$

where $x$ represents each data point (in this case, the latency, power consumption, and network lifetime values for each optimization method), $mean$ is the average value, $n$ is the total number of data points.

## Discussion

The impact of each optimization method—PSO, GR, and the default duty cycle—becomes increasingly significant in long-term network performance and real-world applications, particularly in networks operating over extended periods in diverse environments.

The Particle Swarm Optimization (PSO) method improves latency, power consumption, and network lifetime by iterating toward optimal configurations. This approach offers a balanced solution, making it suitable for dynamic environments like urban or rural areas, where factors such as interference or obstacles may fluctuate. While PSO can adapt to these changes, it introduces additional computational overhead, which may be a challenge in resource-constrained networks.

In contrast, the Golden Ratio (GR) method delivers more pronounced improvements, especially in latency reduction and power savings. By utilizing a deterministic duty cycle distribution, GR ensures stable long-term performance in environments with stable network parameters, such as remote sensing or industrial IoT. Its simplicity and reduced complexity compared to PSO make it more suitable for real-time applications, especially in large-scale networks.

The default duty cycle approach, while simple, serves as a baseline for comparison but tends to perform poorly in scenarios requiring long-term performance. It leads to higher power consumption and shorter network lifetimes, making it less suitable for applications requiring continuous operation over extended periods.

In real-world implementations, PSO requires sophisticated network management to support its iterative process, potentially increasing operational costs. GR, while simpler and more efficient, still requires careful calibration. The default approach, though easier to implement, is suboptimal for long-term use.

The applicability of the proposed Golden Ratio (GR)-based low-duty cycle MAC algorithm to other LoRa bands or configurations remains highly viable, with only a few potential limitations that can be addressed through modifications or further optimizations. While the algorithm was developed and evaluated for the IN 865–867 MHz band, which operates without duty cycle constraints, its core principles are universally applicable to any LoRa band, including those with strict duty cycle limitations like the EU 863–870 MHz ISM band. In these constrained environments, the algorithm can still optimize the duty cycle within the prescribed limits, ensuring efficient operation. The GR approach has the flexibility to adjust its parameters, and its effectiveness does not rely on the absence of duty cycle restrictions but rather on how the duty cycle is managed within the given constraints. Furthermore, while network density and interference may affect performance, the proposed algorithm is designed to handle variable conditions by optimizing sleep periods and transmission scheduling, reducing power consumption and improving latency even in more congested networks. Regarding transmission periods, the algorithm can be extended to dynamically adjust them in response to varying network requirements, making it adaptable to different operational environments. In summary, the algorithm’s robust design ensures that it can be effectively applied across a wide range of LoRa configurations, and any limitations in specific bands or settings can be mitigated through thoughtful adaptations without compromising its overall performance.

In conclusion, PSO and GR offer clear benefits for optimizing network performance, with GR being more suitable for stable, long-term operations, and PSO excelling in dynamic environments. The default method, while easier to implement, is less viable for high-performance, long-term networks. Therefore, optimization methods like PSO and GR are crucial for ensuring efficient and sustainable network operations.

## Conclusion

Among the diverse LPWAN technologies, LoRa operates within the unlicensed ISM band and distinguishes itself as one of the most extensively deployed physical layer technologies, attracting considerable interest in the IoT landscape. However, LoRaWAN’s use of the ISM band introduces duty cycle restrictions, which can impact network performance. In this study, a model for a class A LoRa end-device is developed to evaluate network performance, focusing on latency, power consumption, and network lifetime. Numerical evaluations are conducted for the IN 865–867 band, which operates without duty cycle constraints. To enhance network efficiency, a nature-inspired low-duty cycle MAC algorithm based on the golden ratio (GR) concept is proposed. By aligning duty cycle variables with the golden ratio, the approach optimizes the LoRa network’s duty cycle. The proposed algorithm outperforms the PSO algorithm, achieving a 26% reduction in latency, 12% less power consumption, and a 14% increase in network lifetime compared to the standard duty cycle approach.

While the model effectively integrates key performance parameters using mathematical formulations, it does not explicitly incorporate real-time QoS constraints such as packet delivery rates, reliability, and network stability. Future work will focus on developing a holistic optimization framework that captures the interplay between latency, power consumption, and network lifetime, ensuring a more comprehensive evaluation of LoRa network performance. Additionally, although numerical evaluations validate the model’s effectiveness, extensive simulations and real-world experiments will be conducted in future work to further validate the proposed approach under varying network conditions.

Future research will also explore other metaheuristic algorithms, such as the cuckoo search (CS) algorithm and ant colony optimization (ACO), to further enhance and validate the proposed algorithm’s performance.

## Data Availability

Gagandeep Kaur can be contacted author has to be contacted in case of any queries or requirement of data. Email: gaganksodhi@gmail.com.
